# What Are the Remedies?

**Published:** 1891-12

**Authors:** Robert Farley

**Affiliations:** Phœnixville, Pa.


### WHAT ARE THE REMEDIES?

ROBERT FARLEY, M. D., PHŒNIXVILLE, PA.

The correct answers to the questions given in the October No., at page 393, are as follows: I, Robinia; II, Robinia; III, Robinia; IV, Graph., Nat-m., Puls., Sabin.; V, Calc-p., Cannab., Croc., Cycl., Kali-jod., Merc., Nux-v., Sabina, Thuja; VI, Croc., Mancin., Sang.; VII, Salycyl-ac.; VIII, Salicyl-ac.; IX, Secale; X, Sarsap.; XI, Sarsap.; XII, Sarracenia; XIII, Sarsap.

The following additional questions are now submitted for determination;

XIV. Sensation of cold wet cloths against the thorax in the infra-clavicular regions and in the left infra-mammary region, when out of doors; disappearing when going into the house?

XV. Creeping and crawling under the skin like mice?

XVI. Thinks his head is falling out of bed?

XVII. Thinks there is a devil in his stomach contradicting all he says?

XVIII. Thinks his body is divided and he cannot get the pieces together?

XIX. Feels that she cannot any longer exist?

XX. Thinks his feet are gone and he is walking on his knees.

XXI. Fears to be touched?

XXII. Aversion to being touched?

XXIII. Vertigo arising from epigastrium?

